# Dissipative solitons and backfiring in the electrooxidation of CO on Pt

**DOI:** 10.1038/srep16312

**Published:** 2015-11-10

**Authors:** Philipp R. Bauer, Antoine Bonnefont, Katharina Krischer

**Affiliations:** 1 Non-equilibrium Chemical Physics, Physik-Department, TU München, James-Franck-Str. 1, 85748 Garching, Germany; 2 Institut de Chimie de Strasbourg, UMR7177, CNRS et Université de Strasbourg, 4 rue Blaise Pascal, 67000 Strasbourg, France

## Abstract

Collisions of excitation pulses in dissipative systems lead usually to their annihilation. In this paper, we report electrochemical experiments exhibiting more complex pulse interaction with collision survival and pulse splitting, phenomena that have rarely been observed experimentally and are only poorly understood theoretically. Using spatially resolved *in-situ* Fourier transform infrared spectroscopy (FTIR) in the attenuated total reflection configuration, we monitored reaction pulses during the electrochemical oxidation of CO on Pt thin film electrodes in a flow cell. The system forms quasi-1d pulses that align parallel to the flow and propagate perpendicular to it. The pulses split once in a while, generating a second solitary wave in the backward moving direction. Upon collision, the waves penetrate each other in a soliton-like manner. These unusual pulse dynamics could be reproduced with a 3-component reaction-diffusion-migration model with two inhibitor species, one of them exhibiting a long-range spatial coupling. The simulations shed light on existence criteria of such dissipative solitons.

In excitable media, propagating excitation waves form in a self-organized manner upon a local perturbation of the system, the mechanisms of their formation and propagation being universal. Among the most important examples of such dissipative structures are electrical excitation pulses in nervous tissues, which are indispensable to life for all animate beings with nervous systems^1^. Other prominent examples include the Belousov-Zhabotinsky reaction^2^, the heterogeneously catalyzed CO oxidation on Pt under low pressure conditions^3^,^4^, gas discharge devices^5^, Ca^2+^ ion waves in oocytes^6^, or corroding metals^7^. The theoretical description of an elementary excitation pulse has been successfully accomplished with 2-components activator-inhibitor models, most notably the FitzHugh-Nagumo model, and by now the properties as well as the interactions of such basic pulses are well understood, in particular their annihilation upon collision^8^.

However, there are also rare observations of more complex pulse dynamics. During the oxidation of CO under UHV conditions, solitary wave fragments sometimes continued traveling with their original shape and speed after a collision^9^. Following the nomenclature used for certain wave packets in nonlinear systems with dispersion that emerge unchanged from collisions and can be modeled, for example, by the nonlinear Schrödinger or the Korteweg-de Vries equations, the authors originally characterized these interacting pulses as solitons. To emphasize the different origin of the observed soliton-like behavior of traveling pulses in dissipative systems and classical solitons, nowadays one refers to dissipative solitons and soliton-like behavior rather than just to solitons. Theoretically, dissipative solitons were first predicted to exist in reaction-diffusion systems if the reaction kinetics changes during the collision event^10^. Introducing some local defects in the properties of the catalytic surface in a reaction-diffusion model describing pattern formation during CO oxidation on Pt(110), soliton-like behavior, similar to the one observed in experiments, was obtained when two pulses collided in the vicinity of the defect region^11^. Today it is known that also a third variable may resume the role of the local inhomogeneity, and, in fact, may lead to further intricate interactions of pulses, as for example the splitting of pulses, also called backfiring, the merging of two pulses or the formation of a bound pulse state^12^,^13^,^14^,^15^,^16^,^17^,^18^. Yet, there are still many theoretical questions, and experimental examples of complex pulse interactions remain rare.

In this article, we report the occurrence of soliton-like pulses and backfiring during the electrochemical oxidation of CO on a Pt thin film electrode in a flow cell. The dynamics is reproduced with a 3-component reaction-diffusion-migration system, elucidating the role of the long-range migration coupling for the unusual pulse interaction. The electrooxidation of CO is a prototypical electrocatalytic reaction, which served to elaborate concepts in electrocatalysis^19^,^20^. At the same time it is of significant applied interest, especially in the context of electrode poisoning by adsorbed CO molecules in low temperature fuel cells. In this respect, its dynamical behavior in flow cells with active transport of reactants is of particular interest. For first investigations in this direction see^21^,^22^,^23^.

## Results and Discussion

### Experimental

With a rotating disk electrode or a flow cell, cyclic voltammograms of bulk CO oxidation on Pt electrodes exhibit bistability between a CO covered, poisoned state with negligible reaction rate and a high-current state with diminishing CO coverage in a certain potential region^24^. The positive feedback necessary for any bistability arises from the interaction of the Langmuir-Hinshelwood mechanism governing the reaction mechanism of CO electrooxidation and mass transport limitation. Strongly adsorbing anions, such as Br^−^ or Cl^−^ ions, introduce a negative feedback to the system, rendering it excitable or oscillatory in some parameter ranges^25^,^26^,^27^. The latter system was further investigated in the present study.

**Figure 1:**
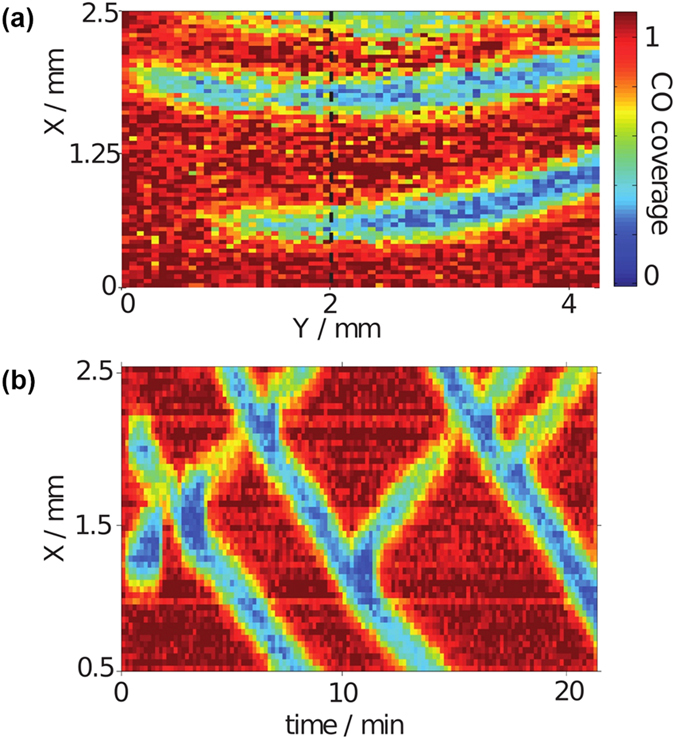
Traveling excitation pulses during CO oxidation on Pt in the presence of Br^−^ ions. (**a**) CO coverage map of the entire visible area of the electrode. (**b**) 1d cut at y ≈ 2 mm, averaged over about 0.5 mm, versus time. Electrolyte: 10 mM H_2_SO_4_, 0.5 *μ*M KBr. Applied voltage 815 mV vs. RHE.

We employed spatially resolved attenuated total reflection (ATR) Fourier transform infrared (FTIR) spectroscopy^28^,^29^ (also known as surface enhanced IR absorption spectroscopy, SEIRAS) to monitor the CO coverage *in-situ* in a flow cell as shown in Fig. 4 in the methods section below, where also experimental details are given. Figure 1(top) depicts a snapshot of the CO coverage on part of the Pt film electrode during CO oxidation in a CO saturated electrolyte containing 0.5 *μ*M Br^−^ ions under potentiostatic conditions. Red indicates a high CO coverage, blue a surface nearly free of CO; the electrolyte flows past the electrode from left to right. The snapshot shows finger-like regions nearly free of adsorbed CO molecules which are aligned parallel to the flow. These reactive areas emerged from the downstream (right) end of the image and spread fast to the left. Once formed, they traveled slowly upwards or downwards, i.e., in the direction perpendicular to the flow with approximately constant shape. The temporal evolution of a cross section through the CO coverage image perpendicular to the electrolyte flow is displayed in Fig. 1(bottom). The 2d spatio-temporal evolution can be viewed in the Supplementary video, where additionally also time series of the total current and the spatially averaged CO coverage are depicted.

Several features can be seen. First, the pulses move with a constant velocity of about 3 *μ*m/s, which is about four orders of magnitude slower than the flow speed along the electrode of about 2 cm/s. This suggests that the fast formation of the reactive fingers parallel to the flow is strongly influenced by advection while the motion perpendicular to the flow is governed by spatial coupling through diffusion and possibly also the electric field but independent of the electrolyte flow.

Second, there are upward and downward moving pulses which penetrate each other upon collision. Hence, we witness here an example of soliton-like behavior in a dissipative system. Three of such pulse interaction events can be seen in Fig. 1(bottom). It is worth mentioning that in the experimental parameter region in which we observed pulses, all pulses survived the collision with another pulse. This is in contrast to the other experiments referenced above where most of the pulse interactions lead to annihilation and only once in a while was a more complex interaction pattern observed^9^,^13^.

Third, as can be seen in Fig 1(bottom) at *t* ≈ 11 and 18 min, a reactive pulse can also split, sending out a finger-like structure in the backward direction. This backfiring is as typical as pulse penetration in our electrochemical system.

To summarize the experimental results, the flow of the electrolyte leads to the alignment of excitation pulses parallel to it. This results in the unusual observation of the spontaneous emergence and the propagation of plane pulses in a two-dimensional active medium, reducing the interaction dynamics of the pulses to one spatial dimension. These quasi-1d pulses exhibit two peculiar properties: They interpenetrate each other upon collision in a soliton-like manner and they may split, sending out a plane pulse with identical speed and shape in the reverse direction.

### Simulations

Next, let us turn to the mathematical modeling. In ref. 27, a set of ordinary differential equations was introduced that captured the global behavior of the total current in the bistable and oscillatory region of CO electrooxidation in the presence of halides in large parameter regions. Here, we consider essentially a spatially extended version of this model. Since the phenomena we are aiming to model are quasi-1d, we only consider the extension of the electrode in one dimension, which we denote by *x*. The temporal evolution of the chemical subsystem, consisting of Θ_CO_, the CO coverage of the electrode, and Θ_X_, the anion coverage is then given by


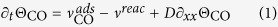



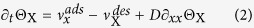


with


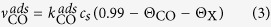



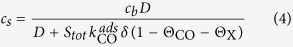



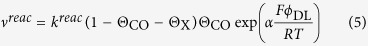



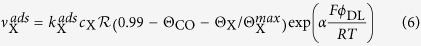



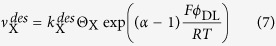


Eq. 1 takes into account that the local CO coverage may change due to adsorption, with the adsorption rate, 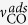, reaction with rate *ν*^*reac*^ and diffusion. 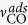 depends on the number of free surface sites and the concentration of CO in the reaction plane, *c*_*s*_. The algebraic equation 4 for the latter results from adiabatic elimination, which keeps the number of variables at a minimum while still capturing the dynamics qualitatively^27^. Note that this would not be the case when ignoring changes of *c*_*s*_ completely, i.e., when treating *c*_*s*_ as a constant (e.g., at the value of the CO bulk concentration, *c*_*b*_). Furthermore, with this ansatz, we also neglect the important lateral diffusion of CO in the electrolyte. This is compensated in part by taking for *D* the value of the diffusion coefficient of CO in aqueous electrolyte. For the reaction rate *ν*^*reac*^ it has been assumed that the OH coverage remains small at all times such that one does not have to take into account its temporal evolution explicitly but can express the reaction rate through the product of the number of free surface sites and Θ_CO_. As shown in ref. 30, this simplification preserves all dynamical features and gives even semi-quantitative results.

The anion coverage is limited to 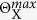. Note the asymmetric inhibition of CO and anion adsorption by adsorbed anions. The latter makes the use of the ramp function 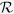 in the adsorption term of the anions necessary, which prevents negative adsorption rates on highly covered surfaces. Finally, the reaction current density is determined by the rates of the charge transfer steps according to


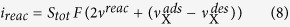


The meaning of all other parameters and their values used in the simulations are given in Table 1. At *x* = 0 and *x* = *L* zero-flux boundary conditions were used.

The system of equations 1, 2 constitutes a reaction-diffusion system of the activator-inhibitor type, with Θ_CO_ being the activator and Θ_X_ the inhibitor. For appropriate values of the parameters, traveling pulse solutions are obtained, which annihilate upon collision. As in the experiments (*cf*. Fig. 1), in the excited regions the CO coverage is low and the current density large while the refractory as well as the excitable rest state are characterized by low local current densities and high CO coverages.

The large difference in local current densities leads already in only moderately conducting electrolytes to a variation of the potential drops across the double layer at the working electrode, i.e., to spatial variations of the effective electrode potential, *ϕ*_DL_. Therefore, a better description is obtained when equations 1, 2 are augmented by an equation for the evolution of *ϕ*_DL_, which results from a local charge balance


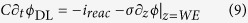


*z* = *WE* is a position at the working electrode, and the second term describes the migration current entering the double layer region. The migration current depends on the normal component of the electric field at the electrode, which is expressed through the electrostatic potential in the electrolyte *ϕ*(*x*, *z*), where *z* is the spatial coordinate perpendicular to the electrode. We model the electrolyte as a two-dimensional, electroneutral medium which obeys Laplace’s equation ∂_*xx*_*ϕ* + ∂_*zz*_*ϕ* = 0, subject to the boundary conditions ∂_*x*_*ϕ*|_*x*=0_ = ∂_*x*_*ϕ*|_*x*=*L*_ = 0, *ϕ*|_*z*=0_ = 0, *ϕ*|_*z*=*WE*_ = −*ϕ*_DL_. *ϕ* and *ϕ*_DL_ are linked to each other through the potentiostatic control condition *U* = *ϕ*_DL_ + *ϕ*|_*z*=*WE*_, whereby it is assumed that the reference electrode is located in the equipotential plane at *z* = 0. Since *ϕ*_DL_ is the only time-dependent boundary condition of Laplace’s equation, Eq. 9 is a closed expression.

**Figure 2:**
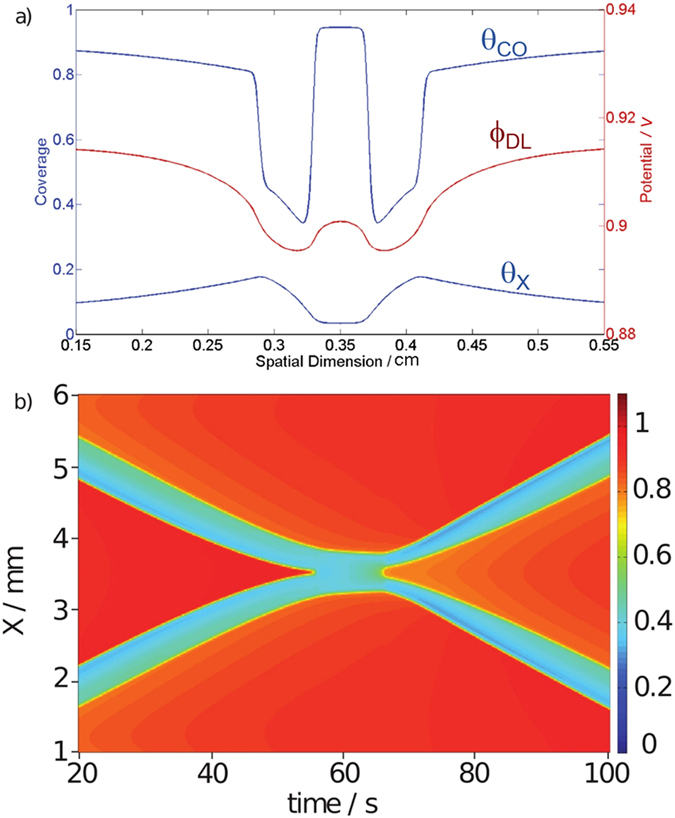
Soliton-like behavior in the 1d mathematical model. (**a**) Spatial profiles of the three variables at *t* = 40 s. Upper profile: CO coverage Θ_CO_, middle profile: electrode potential *ϕ*_DL_, bottom profile: anion coverage Θ_X_. (**b**) CO coverage Θ_CO_ versus time. The parameter values are given in Table 1.

As can be seen in Fig. 2b, when incorporating the electrode potential as additional variable, the model indeed predicts that in certain parameter ranges excitation pulses might survive a collision, thus reproducing soliton-like interactions in a dissipative system. The underlying mechanism can be understood with the profiles taken at *t* = 40 s (Fig. 2a). In the CO (top line) and anion coverage (bottom line), the two pulses seem still to be unaffected from the presence of each other. This can best be seen in the middle of the domain, where the coverages of CO and anions are still close to their values at the rest state. In contrast, the profile of the electrode potential exhibits a value much lower than the one of the rest state in between the approaching pulses because it exhibits a spatial long-ranged coupling: the lower value in the excited region leads to lower electrode potentials in an extended region around the traveling wave.

**Figure 3:**
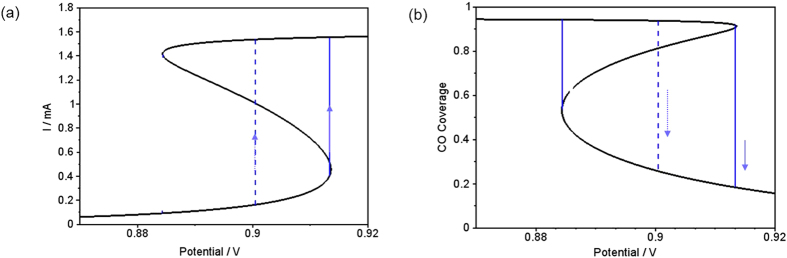
Calculated current density vs. electrode potential (a) and corresponding CO coverage vs. electrode potential (b) at a constant anion coverage Θ_X_ = 0.0427, which is the value at the rest state of the conditions in Fig. 2. The solid vertical line mimics qualitatively an excursion from the rest state to the excitable state of pulse-pulse interaction. The dashed line indicates how this excitation is shifted if the double layer potential is lowered due to the interaction of two pulses.

It is this interaction of the long-range coupling of the electrode potential with the activator-inhibitor sub-system that makes the occurrence of dissipative solitons possible: A lower value of the electrode potential in front of the leading edge of the pulse slows down its propagation velocity. This is illustrated in Fig. 3, where a stationary current potential curve (a) is plotted together with the corresponding stationary CO coverage of the electrode (b). Here, the anion coverage was set to a constant value, more precisely to its value in the rest state, since we are concerned with the excitation from the rest to the excited state at different electrode potentials. The anion coverage becomes only important once the system is excited, its increasing value driving the system eventually back to the low current branch. The rest state is on the low-current branch of the current-potential curve, the excited state on the high-current branch. In this representation it is obvious that a lower value of the electrode potential shifts the system towards a higher excitation threshold, and thus causes more slowly moving pulses. The lower pulse velocity goes along with a decreased anion concentration in the pulse tails, making eventually an excitation at the backsides of the pulses possible. Thus, while the system is undergoing the transition to the refractory state with a high anion concentration in the middle of the collision region, the decreased anion concentration at its tail facilitates the re-excitation of the system at the borders of the bound state, from where two oppositely moving pulses are sent out. Although in the experiments the prolonged interaction time of the pulses cannot be discerned (*cf*. Fig. 1b), the fact that the soliton-like behavior was observed at low concentration of the base electrolyte, where variations of the electrode potential occur and thus the long-range coupling through the electric potential is present, supports the proposed mechanism.

One can easily construct a similar scenario that leads to backfiring. In fact, changing the excitablity in the photosensitive BZ reaction for a certain time by a pulse-like change in illumination intensity was shown to lead to backfiring^31^. The change in illumination intensity has a similar effect as a change of the electrode potential in our system, with the difference that the latter is self-organized while the change of the illumination was controlled from outside. Yet, in our simulations we never observed soliton-like behavior and backfiring for identical parameter values. Backfiring was readily obtained at slightly larger values of the applied potential where, however, the reaction dynamics was oscillatory. Consequently, also the widths of the traveling waves were pulsating in time.

## Conclusions

In conclusion, we have demonstrated that the survival of a collision of excitation pulses occurs in a purely self-organized manner in an electrochemical experiment, without the existence of either inhomogeneities in space nor with man-made changes of a parameter in time. The key ingredient for pulse survival is the interaction of an activator-inhibitor system with a third component with long-range spatial coupling. In electrochemical systems, the electrostatic potential of the electrolyte mediates a long-range coupling of different positions in space, and it is likely that other electrochemical examples with similar pulse dynamics will be found. However, the electric potential is also in other pattern forming dissipative systems a natural variable, such as in semiconductor devices, gas discharge cells or nervous systems. Since a 1/*r* long-range interaction (*r* being the distance to a reference point) is inherent to the electrostatic potential, it can be expected that unusual pulse interactions will also be observed in other environments. Their possible occurrence during nervous signal transmission should be actively tested since this would have tremendous impact on how information processing can be achieved. Moreover, our studies also point to important, open theoretical questions, such as the formulation of a minimal prototypical model as well as more stringent criteria of the occurrence of dissipative soliton-like pulses, the investigation of bifurcations between different pulse interaction events, or a universal and comprehensive classification of them.

## Methods

### Experimental

**Figure 4:**
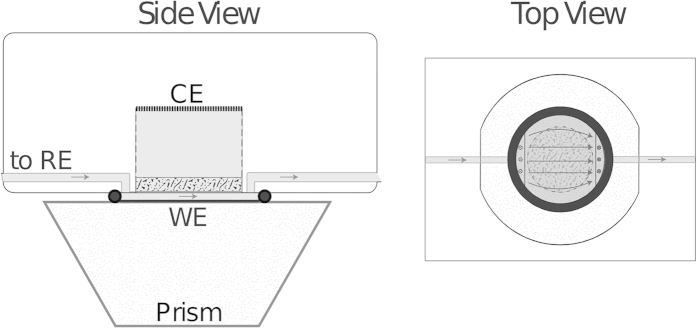
Sketch of the flow cell. Gray areas indicate electrolyte tubes and cell compartment, the arrows mark the flow direction of the electrolyte. Between the WE and the CE compartment was a glass frit that limited the electrolyte flow to a 200 *μ*m thick channel. The Si prism served as IR transparent dielectric for the ATR FTIR measurements. The IR beam was passed through one inclined face and totally reflected at the Si|WE|electrolyte interface.

The experiments were carried out in a flow cell as depicted in Fig. 4 in a side and top view^28^. The cell as well as the tubings (inner diameter: 1.5 mm) were made of PTFE (polytetrafluoroethylene) which was cleaned in a 1:1 mixture of H_2_SO_4_ (96%, p.a, Merck) and H_2_O_2_ (30%, p.a, Merck) and rinsed several times in ultrapure water (Millipore, milliQ). The electrolyte first passed the Hg|Hg_2_SO_4_ reference electrode and then entered the reaction chamber through 3 small intake holes. A 3 mm thick glass frit (P40) limited the flow channel thickness to 0.2 mm and established a conductive connection to the cylindrical counter electrode (CE) compartment of 1 cm height opposing the working electrode (WE). The CE was made of Pt and the electrolyte flow was 3 ml/min.

The WE was a thin Pt film deposited in a two-step process on a Si prism. Prior to film deposition, the prism was ground, polished and cleaned with the RCA method^32^. On the pretreated Si prism, first a Au layer was chemically deposited from sodium tetra chloroaureate(III) solution and HF as reducing agent for 60 s at 60 °C as described in ref. 33. The final platinum deposition was carried out with potassium tetranitroplatinate solution according to the recipe in ref. 34. This two-step process resulted in films with enhanced IR absorption and better adhesion compared to Pt films that were directly deposited on the Si prism according to ref. 34 while exhibiting the electrochemical properties of polycrystalline Pt. The area of deposition was 5 cm^2^ but the reactive geometric area was limited to 1.1 cm^2^ by an o-ring.

For the FTIR measurements, a spatially resolved MCT detector (Bruker FPA, 64 × 64 pixels) with 2.5 mm × 2.5 mm detector area and 1:1 optics is used in an ATR setup as described in refs 28,29. The spectral resolution was set to 8 cm^−1^ and the mean value of five interferograms were used to obtain one coverage map. A long wave pass filter (HP 2340 cm^−1^) improved the acquisition time. The time between each spatial snapshot was 10 s, the collection of the data required 6s. For the calculation of absorbance spectra, a few background spectra with marignial CO coverage were obtained at reactive potentials of 1050 mV. Complementary absorbance spectra with maximal CO coverage were acquired after waiting for one minute at 550 mV and used for CO normalization. To reduce the noise in the background and normalization spectra, each spectrum was obtained from 50 interferograms.

### Simulations

The system of eqs. 1, 2, 9 was solved numerically using a pseudo-spectral code with 513 modes for each variable. For technical details see ref. 35. The numerical time stepping was performed with the SUNDIALS CVode package. All parameters used for the simulations are given in Table 1.

## Additional Information

**How to cite this article**: Bauer, P. R. *et al*.Dissipative solitons and backfiring in the electrooxidation of CO on Pt. *Sci. Rep*.**5**, 16312; doi: 10.1038/srep16312 (2015).

## Supplementary Material

Supplementary Information

Supplementary Video S1

## Figures and Tables

**Table 1:** Meaning of the parameters entering the model and their values used in the simulations.

Meaning of model parameters	Symbol	Value
Potentiostatic control	*U*_0_	1.02 V
Conductivity	*σ*	3 mS cm^−1^
Double layer capacitance	*C*_*Dl*_	2 × 10^−5^ F cm^−2^
Bulk CO concentration	*c*_*b*_	10^−6^ mol cm^−3^ = 1 mM
Anion concentration	*c*_*x*_	10^−9^ mol cm^−3^ = 1 μM
Reactive surface site density	*S*_*tot*_	2 × 10^−9^ mol cm^−2^
Diffusion layer thickness	*δ*	16.44 μm
Transfer coefficient	*α*	0.5
Maximum anion coverage	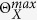	0.8
Anion adsorption rate constant	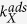	1 cm^3^ s^−1 ^ mol^−1^
Anion desorption rate constant	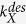	10^6^ s^−1^
CO adsorption rate constant	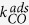	5 × 10^7^ cm^3^ s^−1^mol^−1^
Reaction rate constant	*k*^*reac*^	6 × 10^−7^ s^−1^
Diffusion coefficient	*D*	1.5 × 10^−5^ cm^2^ s^−1^
System length	*L*_*X*_	0.8 cm
Electrode distance	*L*_*Z*_	0.8 cm
Faraday constant/(gas constant × temperature)	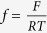	38.7 V^−1^
Faraday constant	*F*	96485 C mol^−1^

## References

[b1] Izhikevich E. M. & FitzHugh R. Scholarpedia 1, 1349 (2006).

[b2] Epstein I. R. & Showalter K. Nonlinear chemical dynamics: Oscillations, patterns, and chaos. J. Phys. Chem. 100, 13132–13147 (1996).

[b3] Ertl G. Oscillatory kinetics and spatio-temporal self-organization in reactions at solid surfaces. Science 254, 1750–1755 (1991). 17829239 10.1126/science.254.5039.1750

[b4] Ertl G. & Rotermund H. H. Spatiotemporal pattern formation in reactions at surfaces. Current Opinion in Solid State and Materials Science. 1, 617–621 (1996).

[b5] Purwins H. G. Self-organized patterns in gas-discharge: particle-like behavior and dissipative solitons. AIP Conference Proceedings. 993, 67–74 (2008).

[b6] Lechleiter J., Girard S., Peralta E. & Clapham D. Spiral calcium wave propagation and annihilation in Xenopus laevis oocytes. Science, 252, 123–126 (1991). 2011747 10.1126/science.2011747

[b7] Kiss I. Z., Nagy T. & Gaspar V. Dynamical instabilities in electrochemical processes. *in* Solid State Electrochemistry II. [Kharton V. V. (ed.)] (Wiley-VCH Verlag, Weinheim, 2011).

[b8] Mikhailov A. S. Foundations of Synergetics I: Distributed Active Systems. (Springer, Berlin, 1991).

[b9] Rotermund H. H., Jakubith S., Von Oertzen A. & Ertl G. Solitons in a surface reaction. Phys. Rev. Lett., 66, 3083–3086 (1991). 10043694 10.1103/PhysRevLett.66.3083

[b10] Tuckwell H. C. Solitons in a reaction-diffusion systems. Science. 205, 493–495 (1979). 17758790 10.1126/science.205.4405.493

[b11] Baer M., Eiswirth M., Rotermund H. H. & Ertl G. Solitary wave phenomena in an excitable surface reaction. Phys. Rev. Lett. 69, 945–948 (1992). 10.1103/PhysRevLett.69.945 10047075

[b12] Purwins H.-G., Bödeker H. U. & Amiranashvili Sh. Dissipative Solitons. Adv. in Physics. 59, 485–701 (2010).

[b13] Von Oertzen A., Mikhailov A. S., Rotermund H. H. & Ertl G. Subsurface oxygen in the CO oxidation reaction on Pt(110): experiments and modeling. J. Phys. Chem. B, 102, 4966–4981 (1998).

[b14] Bordygov G. & Engel H. Anomalous pulse interaction in dissipative media. Chaos, 18, 026104 (2008). 18601506 10.1063/1.2943307

[b15] Wang J. & Mann I. Backfiring and nonannihilation collisions in the Belousov-Zhabotinsky medium. J. Chem. Phys. 11, 7924–7930 (2003).

[b16] Argentina M., Rudzick O. & Velarde M. G. On the backfiring instability. Chaos, 14, 777–783 (2004). 15446988 10.1063/1.1784911

[b17] Manz M. & Steinbock O. Propagation failures, breathing pulses, and backfiring in an excitable reaction-diffusion system. Chaos. 16, 037112 (2006). 17014246 10.1063/1.2266993

[b18] Descalzi O. & Brand H. R. Class of compound dissipative solitions as a result of collisions in one and two spatial dimensions. Phys. Rev. E. 90, 020901 (2014). 10.1103/PhysRevE.90.020901 25215679

[b19] Markovic N. M. & Ross P. Surface science studies of model fuel cell electrocatalysts. Surf. Sci. Reports. 45, 117 (2002).

[b20] Lai S. C. S., Lebedeva N. P., Housmans T. H. M. & Koper M. T. M. Mechanisms of carbon monoxide and methanol oxidation at single-crystal electrodes. Top. Catal. 46, 320–333 (2007).

[b21] Hanke-Rauschenbach R., Kirsch S., Kelling R., Weinzierl C. & Sundmacher K. Oscillations and pattern formation in a PEM fuel cell with Pt/Ru anode exposed to H2/CO mixtures. J. Electrochem. Soc. 157, B1512–B1528 (2010).

[b22] Kirsch S., Hanke-Rauschenbach R. & Sundmacher K. Analysis of spatio-temporal pattern formation in a PEM fuel cell with Pt/Ru anode exposed to H2/CO mixtures. J. Electrochem. Soc. 158, B44–B5 (2011).

[b23] Kirsch S., Hanke-Rauschenbach R., Stein B., Kraume R. & Sundmacher K. The electrooxidation of H2, CO in a model PEM fuel cell: oscillations, chaos, pulses. J. Electrochem. Soc., 160, F436–F446 (2013).

[b24] Koper M. T. M., Schmidt T. J., Markovic N. M. & Ross P. N. Potential oscillations and S-shaped polarization curve in the continuous electro-oxidation of CO on platinum single-crystal electrodes. J. Phys. Chem. B. 105, 8381–8386 (2001).

[b25] Malkhandi S., Bonnefont A. & Krischer K. Strictly potentiostatic current oscillations during bulk CO electro-oxidation on platinum in the presence of inhibiting anions. Electrochem. Comm. 7, 710–716 (2005).

[b26] Malkhandi S., Bonnefont A. & Krischer K. Dynamic instabilities during the continuous electro-oxidation of CO on poly- and single crystalline Pt electrodes. Surf. Sci. 603, 1646–1651 (2009).

[b27] Malkhandi S., Bonnefont A. & Krischer K. Mechanistic aspects of oscillations during CO electrooxidation on Pt in the presence of anions: Experiments and simulations. Catal. Today. 202, 144–153 (2013).

[b28] Morschl R., Bolten J., Bonnefont A. & Krischer K. Pattern formation during CO electrooxidation on thin Pt films studied with spatially resolved infrared absorption spectroscopy. J. Phys. Chem. C. 112, 9548–9551 (2008).

[b29] Bauer P. R., Bonnefont A. & Krischer K. Spatially resolved ATR-FTIRS study of the formation of macroscopic domains and microislands during CO electrooxidation on Pt. ChemPhysChem. 11, 3002–3010 (2010). 20715270 10.1002/cphc.201000301

[b30] Zhang D., Deutschmann O., Seidel Y. E. & Behm. R. J. Interaction of mass transport and reaction kinetics during electrocatalytic CO oxidation in a thin-layer flow cell. J. Phys. Chem. C. 115, 468–478 (2011).

[b31] Munuzuri A. P. & Perez-Villar V. Splitting of autowaves in an active medium. Phys. Rev. Lett. 79, 1941–1944 (1997).

[b32] Kern W. & Puotinen D. A. Cleaning solutions based on hydrogen peroxide for use in silicon semiconductor technology. RCA Rev. 31, 187–206 (1970).

[b33] Miyake H., Ye S. & Osawa M. Electroless deposition of gold thin films on silicon for surface-enhanced infrared spectroelectrochemistry. Electrochem. Comm. 4, 973–977 (2002).

[b34] Miki A., Ye S., Senzaki T. & Osawa M. Surface-enhanced infrared study of catalytic electrooxidation of formaldehyde, methyl formate, and dimethoxymethane on platinum electrodes in acidic solution. J. Electroanal. Chem. 563, 23–31 (2004).

[b35] Plenge F., Li Y. J. & Krischer K. Spatial bifurcations in the generic N-NDR electrochemical oscillator with negative global coupling: Theory and surface plasmon experiments. J. Phys. Chem. B. 108, 14255–14264 (2004).

