# Supplementary material for: Dissipative solitons and backfiring in the electrooxidation of CO on Pt
Source: Sci Rep. 2015 Nov 10;5:16312. doi: 10.1038/srep16312 (PMC4639791; doi:10.1038/srep16312)
Supplement: Supplementary Information [file srep16312-s1.pdf]

Dissipative solitons and backfiring in the electrooxidation of CO on Pt

Philipp R. Bauer,[1] Antoine Bonnefont,[2] and Katharina Krischer[1]

[1]Non-equilibrium Chemical Physics, Physik-Department,
TU München, James-Franck-Str. 1, 85748 Garching, Germany
[2]Institut de Chimie de Strasbourg, UMR7177, CNRS et Université de Strasbourg,
4 rue Blaise Pascal, 67000 Strasbourg, France

The video shows the 2d spatio-temporal evolution of traveling excitation waves exhibiting penetration and backfiring together with time series of the total current (blue line) and the spatially averaged CO coverage (green line). Experimental conditions are the same as those of Fig. 1.
